# Iron status at early pregnancy is associated with infectious respiratory and gastric illness in women receiving routine iron supplementation: the NuPED prospective cohort

**DOI:** 10.1186/s12884-025-07786-8

**Published:** 2025-06-04

**Authors:** Caylin Goodchild, Elizabeth A. Symington, Jeannine Baumgartner, Lizelle Zandberg, Amy J. Wise, Cornelius M. Smuts, Linda Malan

**Affiliations:** 1 Centre of Excellence for Nutrition, North-West University, Potchefstroom, South Africa; 2 Department of Life and Consumer Sciences, University of South Africa, Johannesburg, South Africa; 3 Department of Nutritional Sciences, King’s College London, London, UK; 4 Department of Obstetrics and Gynaecology, University of the Witwatersrand, Johannesburg, South Africa; 5 Empilweni Services and Research Unit, University of the Witwatersrand, Johannesburg, South Africa

**Keywords:** Anaemia, Infectious morbidity, Iron supplementation, Pregnancy

## Abstract

**Background:**

Antenatal iron deficiency (ID) and anaemia, but also elevated ferritin and haemoglobin (Hb) have been associated with morbidity during pregnancy. In South Africa, pregnant women receive routine iron supplementation for anaemia prevention regardless of iron status. Our aim was to assess whether iron status at early pregnancy is associated with infectious morbidity and symptoms during pregnancy.

**Methods:**

This prospective cohort was conducted in 250 pregnant women at a public maternal and child hospital in Johannesburg, South Africa. Biomarkers of maternal iron status at < 18 weeks’ gestation were measured. Women kept a symptoms diary throughout pregnancy. Associations were determined using multivariable regression models.

**Results:**

ID women had 2.6 times greater odds for experiencing gastric illness (OR: 2.642, 95% CI: 1.116, 6.255, *p* = 0.027). Anaemic women (Hb < 10.5 g/dL) tended to have double the duration of respiratory illness [median 15.5 (5.0, 31.0) days] compared to non-anaemic women [median 8.0 (6.0, 12.1) days], (β: 0.167, 95% CI: -0.007, 0.342, *p* = 0.060) and had more incidences of vomiting throughout pregnancy (*p* = 0.028). In the partially adjusted models, iron sufficient erythropoiesis (non-IDE) women tended to have 2.3 times increased odds for respiratory illness (OR: 2.314, 95% CI: 0.939, 5.701, *p* = 0.068) and there were more incidences of fever during pregnancy in the non-IDE group (*p* = 0.006).

**Conclusion:**

Anaemic and ID pregnant women in this largely overweight population, receiving ~ 55 mg iron daily, experience more and longer infectious morbidity, potentially related to poor iron absorption. However, although presenting with weaker evidence, iron-sufficient erythropoiesis women at early pregnancy receiving the same routine iron supplementation may have twice the risk to contract infectious respiratory illness than IDE women during pregnancy.

**Supplementary Information:**

The online version contains supplementary material available at 10.1186/s12884-025-07786-8.

## Background

Anaemia during pregnancy is present in 37% of women worldwide. Nutrition-related anaemia is predominantly caused by iron deficiency (ID) [1]. The long-term prevalence (1969–2020) of anaemia in pregnant women in South Africa is 31% (95% CI, 23–40%), with a small sample from the South Africa Demographic and Health Survey 2017 demonstrating a prevalence of 39.1% (95% CI 36%–42.2%) [2, 3]. These agree with some smaller studies indicating that 29% to 43% of expectant mothers are anaemic [4]. In the current study, the Nutrition during Pregnancy and Early Development (NuPED) cohort (2016–2017) in Gauteng Province, urban South Africa, anaemia was 29% and ID was 15% in early pregnancy [5].

Anaemia and ID during pregnancy are more common compared to non-pregnant women, even when accounting for haemodilution, as the growing foetus and the increase in maternal erythrocyte mass requires additional iron [6]. Prevention of anaemia and ID is paramount to avoid negative pregnancy outcomes for mother and child. These include increased maternal sickness, low birthweight and small-for-gestational age, preterm birth, intrauterine growth retardation, miscarriage, prenatal depression, heart failure and even maternal death [7, 8]. Children born to mothers with ID have been found to have memory and learning difficulties which continue into adulthood [9].

To prevent anaemia and ID during pregnancy, the World Health Organisation (WHO) recommendation on multiple micronutrient supplement (MMS) has recently changed from “not recommended” to “recommended in the context of rigorous research” in pregnant women. The recommended MMS contains 30 mg of iron [10]. In addition, it has recently been found, with a novel stable isotope technique conducted in free-living individuals, that iron loss increased three-fold in Swiss women of reproductive age and also increased in Gambian toddlers, but not in Beninese women, after supplementation with 50 mg iron as ferrous sulphate, compared to before iron supplementation [11]. However, 60 mg iron is still recommended in countries where anaemia is a severe public health concern (≥ 40%) depending on the contribution of ID and other factors [10]. Currently in South Africa, pregnant women routinely are provided with 200 mg ferrous sulphate (~ 55 mg elemental iron) daily (plus calcium and folic acid), regardless of their iron status [12].

There have been concerns about possible adverse effects caused by a high dose of non-haem iron supplementation, specifically in women who are iron replete and women who do not absorb iron sufficiently. It has been found that certain populations, such as people living with obesity, have poor absorption due to chronic low-grade inflammation, and therefore iron supplementation does not improve their iron status as effectively as desired [5, 13, 14]. It has also been shown that infectious morbidity may be increased by iron supplementation [15]. A high maternal iron status has also been associated with infants that are preterm, low birth weight, small-for-gestational age and foetal neurodegeneration [16]. This double-edged sword poses a challenge to managing iron status and may require targeted nutrition interventions.

Considering the concerns regarding iron supplementation in iron replete women and in women with potentially poor absorption, the aim of this study was to determine associations of iron status at early pregnancy (< 18 weeks’ gestation) with infectious morbidity and symptoms during pregnancy in women routinely receiving ~ 55 mg elemental iron supplementation in urban Johannesburg, South Africa. We hypothesised that pregnant women with replete iron status in early pregnancy who receive ~ 55 mg of routine iron supplementation experience a higher incidence of infectious illness compared to iron-deficient pregnant women.

## Methods

### Study design and site

The investigation of maternal morbidity during pregnancy was a secondary objective of the prospective NuPED cohort study, conducted in Johannesburg, South Africa, which is the largest city in the urban Gauteng province. The study protocol was previously published by Symington et al*.* [17]. Briefly, consecutive sampling of 250 pregnant women took place between March 2016 and November 2017 at four primary healthcare clinics in Johannesburg. The clinics fall in the catchment area of a provincial hospital focusing on maternal and paediatric healthcare and delivering more than 10,000 babies annually, where follow-up visits took place.

### Participants

Consecutive sampling was applied whereby all accessible pregnant women at the recruitment sites who consented formed part of the sample. All pregnant women in the waiting areas of the antenatal clinics were informed about the study. Those interested received a study information leaflet and were screened for eligibility individually in a private space upon which a booking date was supplied if eligible. Participants were ensured that participation was voluntary and that participation or non-participation in the study will not affect their clinical care. All participants provided written informed consent before data collection. Data were anonymised by assigning participant numbers. A post hoc power calculation based on multiple linear regression analysis (fixed model, single regression coefficient) showed that a sample size of 125 provided 99% power to detect a medium effect size F2 of 0.15 with a probability of error (alpha) of 5% when including ten predictors.

### Eligibility criteria

Women (*n* = 250) were eligible for the study if they were < 18 weeks pregnant with singleton pregnancies, aged 18–39 years, proficient in local languages, born in South Africa or neighbouring countries, and if they had been residing in Johannesburg for at least 12 months. Women were excluded if they were smoking, reported using illicit drugs, or had previously been diagnosed with a non-communicable disease (namely hypertension, renal disease, diabetes and high cholesterol), an infectious disease (namely hepatitis and tuberculosis), or a serious illness (namely lupus, cancer or psychosis). Due to South Africa’s high prevalence of human immunodeficiency virus (HIV) infection, women living with HIV were included in the study. Eligible women who agreed to participate were followed up three times during pregnancy (< 18 weeks’, 22 weeks’ and 36 weeks’ gestation) at an academic maternal and child hospital’s antenatal clinic.

### Ethical considerations

This study was conducted according to the guidelines laid down in the Declaration of Helsinki and approved by both the Human Research Ethics Committees of the North-West University (NWU-00186–15-A1) and the University of the Witwatersrand (M150968).

### Outcome measurements

The primary outcome was incidence of infectious respiratory illness, and secondary outcomes were 1) incidence of infectious gastric illness, 2) duration of respiratory illness, and 4) duration of gastric illness, throughout pregnancy. An additional explorative outcome was the incidence of individual symptoms, i.e. coughing, diarrhoea, fever, nausea, vomiting, headache and extreme tiredness, during pregnancy. Symptoms were recorded by the women in a symptoms diary from enrolment (< 18 weeks’ gestation) and collected at study site visits on 22- and 36-weeks’ gestation visits. Symptoms listed in the pre-designed diary were coughing, diarrhoea, fever, nausea, vomiting, headache and extreme tiredness. An incidence of respiratory illness was scored if coughing and fever symptoms were present simultaneously for three or more days. An incidence of gastric illness was scored if diarrhoea and fever symptoms were present simultaneously for three or more days. Incidences of gastrointestinal symptoms or respiratory symptoms without fever were not included. New incidences were scored after a two-day symptom free period.

### Exposure measurements

Iron status biomarkers, including ferritin and soluble transferrin receptor (sTfR) and Hb concentrations were measured at enrolment (< 18 weeks’ gestation) and mid-pregnancy (22 weeks’ gestation). Maternal venous blood was drawn into serum and EDTA-coated evacuated tubes. Serum was separated within 1 h after blood draw and stored at −20 °C for a maximum of 14 days until transportation for storage at −80 °C until analysis. Ferritin, sTfR and inflammatory markers (α1-acid glycoprotein [AGP] and C-reactive protein [CRP]) concentrations were measured with the Q-Plex Human Micronutrient Array (7-plex, Quansys Bioscience, Logan, UT, USA) [18]. The inflammatory markers were used to adjust ferritin for inflammation [19]. Iron deficiency (ID) was defined as adjusted ferritin < 15 μg/L [20]. Iron deficiency erythropoiesis (IDE) was defined as sTfR > 8.3 mg/L [21]. Moderate iron stores (MIS) were defined as ferritin ≥ 50 μg/L.

Hb concentrations were analysed with calibrated HemoCue meters (Hb 201 +, Ängelholm, Sweden in venous whole blood samples (20 μL). Due to Johannesburg’s location being 1753 m above sea level [22] Hb values were adjusted for altitude by deducting 0.5 g/dL [23]. Our sample were non-smokers therefore no adjustments were made for smoking. Anaemia was defined as Hb < 10.5 g/dL based on cut-offs per trimester [24] and as Hb < 11 g/dL according to the WHO for comparison purposes [23].

Maternal dietary intake data were collected using both a 24-h recall (24-HR) and a quantified food frequency questionnaire (QFFQ). The 24-HR was used to obtain information regarding nutritional supplement use at < 18-, 22- and 36-weeks’ gestation while the QFFQ data were used to determine iron intake at < 18 weeks’ gestation. Both methods were administered by an interviewer using standardised probing questions. To assist in accurately quantifying portion sizes, common size food containers (such as cups and bowls), standard measuring instruments, and food models were used.

The QFFQ was validated using the Transition and Health during Urbanisation of South Africans (THUSA) study [25]. This QFFQ included a list of approximately 140 typically consumed foods items. Specific questions were asked pertaining to the brand of food, cooking methods, frequency of consumption and the quantity of all food and drink consumed over the past month.

This data were converted to grams per week per food item by registered dietitians/nutritionists, using Condensed Food Composition Tables for South Africa [26] and the South African Medical Research Council (SAMRC) Food Quantities Manual [27]. The total daily dietary iron intake levels were obtained by linking dietary food intake data to the most recent food composition database by the SAMRC. Iron content of fortified foods are included as per the national food fortification programme in the database.

Supplement use reported in the 24-HR was cross-checked using a calendar on which participants recorded supplement use (yes/no). Additionally, at each visit, participants were asked about the brand, type, frequency and amount of all dietary supplements (including routine care supplements) used in the past week. Total iron intake from supplements during pregnancy was calculated.

### Covariates and characteristics

Maternal age, level of education and living standards measurements (LSM) were obtained by use of a questionnaire conducted by an interviewer [28]. Medical files were inspected to obtain parity and HIV status, which was updated at birth. Height, unadjusted weight and mid-upper arm circumference (MUAC) at early pregnancy was measured using the International Society for the Advancement of Kinanthropometry [29, 30]. Body mass index (BMI) was calculated as weight (kg) divided by height (m) squared. Underweight was defined as BMI < 18.5 kg/m^2^, normal weight as 18.5–24.9 kg/m^2^, overweight as 25–29.9 kg/m^2^ and obese as ≥ 30 kg/m^2^. Red blood cell (RBC) omega-3 polyunsaturated fatty acid (n-3 PUFA) composition was measured with gas chromatography mass spectrometry [31].

Gestational age at enrolment was determined using foetal ultrasonography [32]. Covariates were included in the regression models determining the associations of antenatal iron status and anaemia with incidence and duration of infectious morbidity.

### Statistical analysis and data management

Data were analysed with SPSS version 27 (SPSS Inc, Chicago, IL, USA). Raw data were captured in Microsoft Access (Microsoft Corporation, Washington, USA) and 20% of all captured data were randomly checked for correctness. Data were tested for outliers and normality by means of Q-Q plots, histograms and Shapiro–Wilk tests. Normally distributed data are expressed as means ± SD; non-normally distributed data are expressed as medians (25 th, 75 th percentiles). Potential differences in characteristics of pregnant women at enrolment (< 18 weeks’ gestation) by presence of gastric or respiratory illness throughout pregnancy were analysed with Mann–Whitney-U tests for continuous variables, and Chi-square tests for categorical variables.

Associations of maternal iron status at early pregnancy (< 18 weeks’ gestation) with either experiencing gastric/respiratory illness in pregnancy or not were assessed with multivariable logistic regression. Odds ratios (OR) and 95% confidence intervals (CI) were reported. Associations of maternal iron status at early pregnancy with the total number and the duration of incidences were analysed with Poisson regression with a log linear link and ANCOVA, respectively. ß-values and 95% CIs were reported. Longitudinal associations between maternal iron status at early pregnancy with morbidity symptoms during pregnancy were assessed with linear mixed models and Sidak corrections for 18–22 weeks’ gestation (n = 232) and 18–36 weeks'gestation (*n* = 192), respectively. Participants were treated as random effects, and group and time (week of gestation) were assessed separately as fixed effects. We constructed both partially adjusted and fully adjusted regression models to explore the relationship of iron status with infectious morbidity and symptoms. The partially adjusted model included maternal age, parity, HIV status, education, total days monitored for morbidity and height selected a priori based on clinical relevance and literature. The fully adjusted model included all covariates considered potential confounders or associated with the outcome, additionally including MUAC, living standards measure (socio-economic status), gestational age and n-3 PUFA status. Given the small sample size, we present both adjusted models to balance confounder control with concerns about overfitting and model stability. For the linear mixed model analysis, only results for model 2 are shown (Fig. 2). *P* ≤ 0.05 was considered significant and P between 0.05–0.10 to have a tendency toward significance.

## Results

### Study profile

**Fig. 1:**
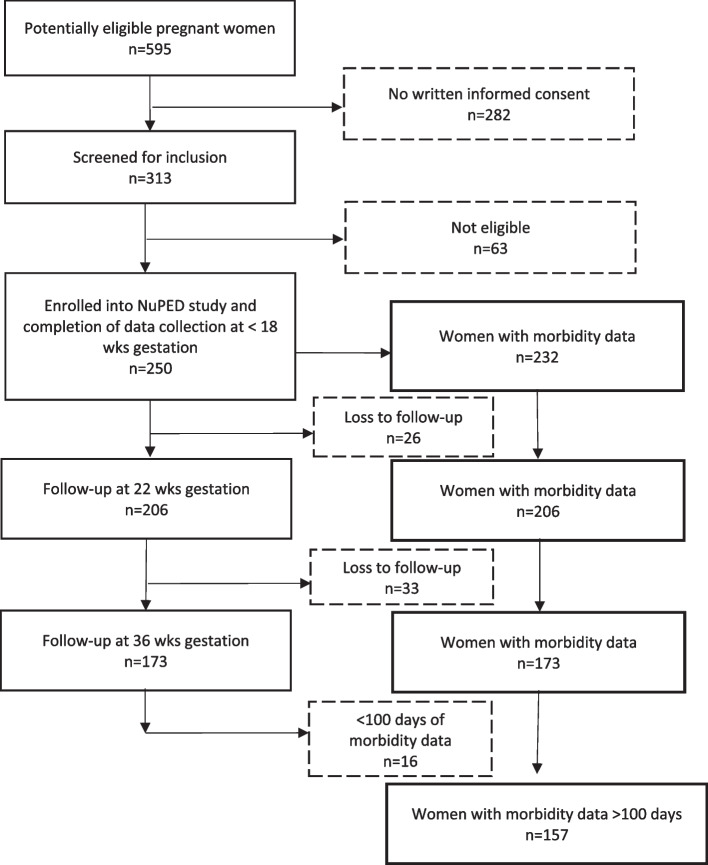
Flowchart of pregnant women included and followed up in the NuPED study and availability of data for analysis in this study

A flowchart of the study, including data availability, is shown in Fig. 1. In total, 232/250 participants had morbidity data, and 78 (33.6%) women experienced infectious respiratory illness (with fever), whereas 47 (20.3%) experienced infectious gastric illness at least once during pregnancy. The duration of data collected ranged from 21- 245 days. Of these 232 women with morbidity data, 167 and 157 participants had data for more than 100 days, from 18–22 weeks’ and 18–36 weeks’ gestation, respectively. Participant characteristics of the 157 participants with > 100 days’ data did not differ from those with missing or < 100 days’ data (Supplementary Table 1).

### Participant characteristics at early pregnancy

A total of 117 (47%) of women had moderate iron stores of ferritin ≥ 50 ug/L after adjusting for inflammation. Eighty-eight percent of the 250 women were of self-identified black-African descent (Table 1). At enrolment, the women’s median age was 27 (24, 32) years and median gestational age was 14 (12, 17) weeks. Most women completed their education in grades 11 and 12 (58%), with 23% continuing their post-school education. Fifty-nine percent of women had a medium LSM score indicating a middle-class living standard. Thirty percent were nulliparous. In total, 33% of women were overweight and 29% obese. A total of 60% of women had an elevated CRP and 26% were HIV positive.

**Table 1:** Characteristics of pregnant women at enrolment (< 18 weeks’ gestation) grouped by women who experienced respiratory and gastric illness throughout pregnancy compared to no illness^a^

**Characteristics**	**Total sample** **(*n* = 249–250)**	**Respiratory illness** **(*n* = 73–78)**	**No respiratory illness** **(n = 144–154)**	**p**	**Gastric illness** **(*n* = 47)**	**No gastric illness** **(*n* = 184–185)**	**p**
	**Median (25 th, 75 th percentile)** **or n (%)**	**Median (25 th, 75 th percentile)** **or n (%)**	**Median (25 th, 75 th percentile)** **or n (%)**		**Median (25 th, 75 th percentile)** **or n (%)**	**Median (25 th, 75 th percentile)** **or n (%)**	
**Age (y)**	27.0 (24.0, 32.0)	28.0 (24.0, 32.0)	27.0 (24.0, 32.0)	0.807	27.0 (23.0, 30.0)	28.0 (24.0, 32.0)	0.192
**Gestational age (wks)**	14.0 (12.0, 17.0)	15.0 (13.0, 16.0)	14.0 (12.0, 16.0)	0.436	13.1 (11.7–16.1)	14.3 (12.1, 16.0)	0.568
**BMI (kg/m**^**2**^**)**	26.3 (23.0, 30.6)	26.7 (23.6, 30.8)	25.9 (22.6, 30.6)	0.203	25.2 (22.5, 28.8)	26.5 (23.1, 31.2)	0.360
**Underweight (< 18.5 kg/m**^**2**^**)**	8 (3)	2 (3)	5 (3)	0.845	1 (2)	6 (3)	0.206
**Normal weight (18.5–24.9 kg/m**^**2**^**)**	89 (36)	25 (32)	58 (38)		21 (45)	62 (34)	
**Overweight (25–29.9 kg/m**^**2**^**)**	81 (33)	27 (35)	47 (31)		15 (32)	59 (32)	
**Obese (≥ 30 kg/m**^**2**^**)**	71 (29)	23 (30)	44 (29)		10 (21)	57 (31)	
**MUAC Ethnicity**	29.9 (27.1, 33.1)	30.1 (27.8, 34.4)	29.8 (27.0, 32.2)	0.102	28.5 (26.5, 30.2)	30.1 (27.5, 33.2)	0.115
**Black African**	219 (88)	66 (85)	138 (90)	0.290	41 (87)	163 (89)	0.779
**Mixed ancestry**	28 (11)	12 (15)	14 (9)		6 (13)	20 (11)	
**White**	1 (0.4)	-	-		0	-	
**Indian**	1 (0.4)	0	1 (1)		0	1 (1)	
**LSM**							
**Low (1–4)**	17 (7)	5 (6)	10 (7)	0.764	6 (13)	9 (5)	0.328
**Medium (5–7)**	148 (59)	45 (58)	96 (62)		25 (53)	116 (63)	
**High (8–10)**	85 (34)	28 (36)	48 (31)		16 (34)	60 (32)	
**Education**							
**Primary school**	9 (4)	4 (5)	5 (3)	0.189	2 (4)	7 (4)	0.414
**Grade 8–10**	37 (15)	14 (18)	21 (14)		11 (23)	24 (13)	
**Grade 11–12**	145 (58)	49 (63)	87 (57)		24 (51)	112 (61)	
**Post-school**	58 (23)	11 (14)	40 (26)		10 (21)	41 (22)	
**Parity**							
**Nulliparous**	74 (30)	17 (22)	51 (33)	0.312	17 (36)	51 (28)	0.826
**Primiparous**	88 (35)	28 (36)	52 (34)		17 (36)	63 (34)	
**Multiparous**	88 (35)	33 (42)	51 (33)		13 (28)	71 (38)	
**HIV status**							
**Positive**	64 (26)	19 (24)	42 (27)	0.634	11 (23)	50 (27)	0.597
**Inflammatory status**							
**CRP (mg/L)**	6.5 (3.1, 14.4)	8.6 (3.7, 18.3)	5.8 (2.7, 12.9)	0.040	5.8 (3.3, 10.4)	7.0 (3.0, 15.8)	0.410
**Elevated CRP (> 5 mg/L)**	149 (60)	54 (69)	84 (55)	0.031	20 (43)	111 (60)	0.942
**RBC PUFA composition (% of total fatty acids)**							
**n-3 LCPUFA**	7.4 (6.4, 8.3)	7.0 (6.0, 8.2)	7.5 (6.6, 8.4)	0.032	6.9 (6.2, 8.0)	7.4 (6.5, 8.4)	0.152
**n-6 LCPUFA**	19.6 (18.3, 20.7)	19.4 (18.0, 20.2)	19.8 (18.4, 20.8)	0.205	19.8 (18.4, 20.4)	19.6 (18.1, 20.7)	0.910

*y* years, *MUAC* mid upper arm circumference, *wks* weeks, *LSM* living standards measure, *BMI* body mass index, *CRP* C-reactive protein, *mg* milligrams, *RBC* red blood cell, *PUFA* polyunsaturated fatty acid *n-3 LCPUFA* omega-3 long-chain polyunsaturated fatty acid, *n-6 LCPUFA* omega-6 long-chain polyunsaturated fatty acid

^a^Differences between groups were analysed with Mann–Whitney-U tests for continuous variables and Chi-square tests for categorical variables. BMI, ethnicity, and education missing one data point

*P* ≤ 0.05 was considered significant

Women who experienced respiratory illness had a higher CRP at enrolment compared to women with no respiratory illness (*p* = 0.040). Elevated baseline CRP was present in 69% compared to 55% of women who did not experience respiratory illness during pregnancy (*p* = 0.031). The RBC n-3 PUFA composition was higher in women with no respiratory illness (*p* = 0.032).

### Dietary and supplemental iron intake

Table 2 shows that there was no difference in estimated total iron supplement intake during pregnancy as well as daily dietary iron intake between the women that experienced respiratory illness and those that experienced no respiratory illness (p = 0.241 and p = 0.565 respectively) nor between the women that experienced gastric illness and those that experienced no gastric illness (p = 0.297 and p = 0.940 respectively).

**Table 2:** Dietary and supplemental iron intake throughout pregnancy grouped by women who experienced respiratory and gastric illness compared to no illness^a^

	**Total sample** **(*n* = 250)**	**Respiratory illness** **(*n* = 73–78)**	**No respiratory illness** **(n = 144–154)**	***P***	**Gastric illness** **(*n* = 47)**	**No gastric illness** **(*n* = 184–185)**	***P***
**Daily dietary iron intake (mg)**	17.9 (13.7, 22.9)	18.2 (13.1, 24.9)	18.0 (14.2, 22.9)	0.565	19.6 (10.4, 24.9)	17.9 (14.0, 23.0)	0.940
**Total iron supplement intake (mg)**	8965 (5775, 10,615)	8855 (5673, 10,313)	9048 (6724, 10,670)	0.241	8855 (3608, 10,395)	9020 (6710, 10,670)	0.297

*mg* milligrams

^a^Differences between groups were analysed with Mann–Whitney, U tests

*P* ≤ 0.05 was considered significant. Data presented as median (25^th^, 75^th^ percentile)

#### Associations of iron status with respiratory and gastric illness

**Fig. 2:**
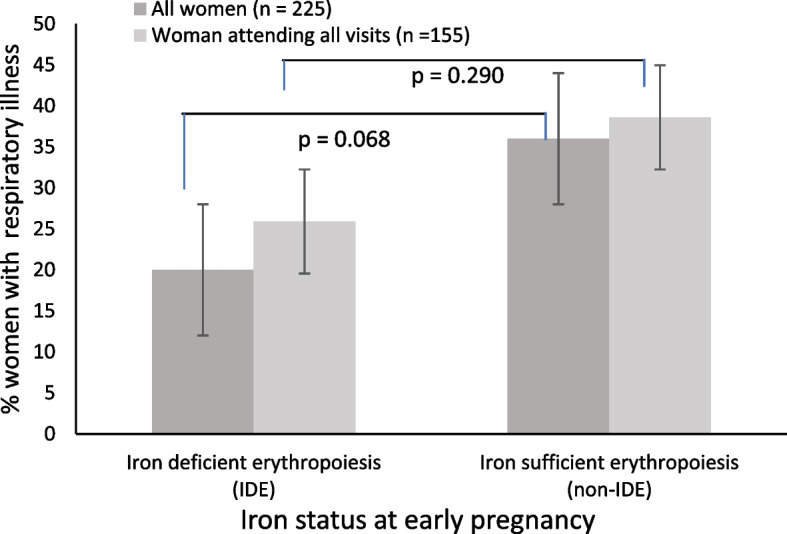
Percentage of iron deficient erythropoiesis IDE compared to iron sufficient erythropoiesis women at early pregnancy experiencing any infectious respiratory illness during pregnancy

Table 3 shows that participants with ID had 2.6 times greater odds for experiencing gastric illness in the fully adjusted model (OR: 2.642, 95% CI: 1.116, 6.255, *p* = 0.027). Women with IDE tended to have 57% reduced odds to experience respiratory illness (OR: 0.432, 95% CI: 0.175, 1.065, *p* = 0.068) in the partially adjusted model (maternal age, parity, HIV, education, total days of morbidity monitored and height). Consequentially, non-IDE (iron sufficient erythropoiesis) women tended to have 2.3 times increased odds for having respiratory illness (OR: 2.314, 95% CI: 0.939, 5.701, *p* = 0.068) in the partially adjusted model depicted in Fig. 2. The IDE association did not maintain statistical significance in the fully adjusted model. In a sensitivity analysis (Supplementary Table 2) in the 157 participants with data for both visits (22 and 36 weeks) and monitored for > 100 days, similar results were found with ID tending to have 2.6 times greater odds for experiencing gastric illness in the fully adjusted model (OR: 2.569, 95% CI: 0.869, 7.596, *p* = 0.088).

**Table 3:** Associations of maternal iron status at early pregnancy with experiencing respiratory or gastric illness at least once during pregnancy (multivariable logistic regression)

**Exposure**			**Respiratory illness**							
	**Case Fraction (%)**	**Control Fraction (%)**	**Model 1**				**Model 2**			
			**OR**	**95% CI**	**P**	**n**	**OR**	**95% CI**	**P**	**n**
Anaemia (Hb < 10.5 g/dL)	15/39 (38.5)	61/187 (32.6)	1.377	0.653, 2.905	0.401	225	1.302	0.595, 2.847	0.508	220
Anaemia (Hb < 11.0 g/dL)	21/66 (31.8)	55/160 (34.4)	0.868	0.459, 2.164	0.663	225	0.832	0.425, 1.630	0.592	220
ID (Fer < 15 μg/L)	10/34 (29.4)	68/198 (34.3)	0.825	0.363, 1.877	0.647	231	0.970	0.413, 2.281	0.945	225
IDE (sTfR > 8.3 mg/L)	7/35 (20.0)	71/197 (36.0)	0.432	0.175, 1.065	0.068	231	0.580	0.227, 1.482	0.255	225
MIS (Fer ≥ 50 μg/L)	41/109 (37.6)	37/123 (30.1)	1.331	0.757, 2.342	0.321	231	1.313	0.718, 2.402	0.377	225
			**Gastric illness**							
			**Model 1**				**Model 2**			
Anaemia (Hb < 10.5 g/dL)	9/39 (23.1)	36/187 (19.3)	1.425	0.603, 3.372	0.420	225	1.396	0.577, 3.375	0.459	220
Anaemia (Hb < 11.0 g/dL)	13/66 (19.7)	32/160 (20.0)	1.016	0.482, 2.140	0.967	225	1.057	0.492, 2.265	0.889	220
ID (Fer < 15 μg/L)	12/34 (35.3)	35/198 (17.7)	2.487	1.089, 5.679	**0.031**	231	2.642	1.116, 6.255	**0.027**	225
IDE (sTfR > 8.3 mg/L)	7/35 (20.0)	40/197 (20.3)	0.988	0.393, 2.481	0.979	231	1.207	0.463, 3.149	0.700	225
MIS (Fer ≥ 50 μg/L)	20/109 (18.3)	27/123 (22.0)	0.805	0.414, 1.566	0.532	231	0.756	0.378, 1.513	0.430	225

*Hb* haemoglobin, *Fer* serum ferritin, *sTfR* soluble transferrin receptor, *ID* iron deficiency, *IDE* iron deficiency erythropoiesis, *MIS* moderate iron stores, *OR* odds ratio, *CI* confidence interval

Associations assessed with multivariable logistic regression, odds ratios and 95% confidence intervals. *P* ≤ 0.05 was considered significant and *P* between 0.05–0.10 to have a tendency toward significance. Model 1 is adjusted for maternal age, parity, HIV, education; total days of morbidity monitored and height; model 2 adjusted additionally for MUAC, living standards measures (socio, economic status), gestational age and RBC n-3 LCPUFA composition

Table 4 shows that there was no association between maternal iron status and total incidences per participant of either respiratory or gastric illness.

**Table 4:** Iron status in association with total incidences per participant of respiratory or gastric illness during pregnancy

Exposure			**Respiratory illness**							
	**Number of incidences**^**a**^		**Model 1**				**Model 2**			
	**Case**	**Control**	**β**	**95% CI**	**P**	**n**	**β**	**95% CI**	**P**	**n**
Anaemia (Hb < 10.5 g/dL)	2.0 (1.0, 3.0)	2.0 (1.0, 2.0)	0.033	−0.221, 0.287	0.799	225	0.033	−0.230, 0.297	0.804	220
Anaemia (Hb < 11.0 g/dL)	2.0 (1.0, 3.0)	2.0 (1.0, 2.0)	0.088	−0.121, 0.297	0.678	225	0.092	−0.123 0.307	0.401	220
ID (Fer < 15 μg/L)	2.0 (1.0, 3.0)	2.0 (1.0, 2.0)	0.012	−0.256, 0.280	0.932	231	0.002	−0.273, 0.277	0.989	225
IDE (sTfR > 8.3 mg/L)	2.0 (1.0, 2.2)	2.0 (1.0, 2.0)	0.003	−0.261, 0.267	0.982	231	−0.023	−0.303, 0.256	0.869	225
MIS (Fer ≥ 50 μg/L)	2.0 (1.0, 2.0)	2.0 (1.0, 2.0)	−0.036	−0.226, 0.155	0.714	231	−0.012	−0.210, 0.186	0.905	225
			**Gastric illness**							
			**Model 1**				**Model 2**			
Anaemia (Hb < 10.5 g/dL)	2.0 (1.0, 5.0)	2.0 (1.0, 3.0)	−0.004	−0.252, 0.243	0.973	225	−0.014	−0.271, 0.244	0.916	220
Anaemia (Hb < 11.0 g/dL)	2.0 (1.0, 2.0)	2.0 (1.0, 3.0)	−0.047	−0.254, 0.160	0.658	225	−0.044	−0.258, 0.169	0.684	220
ID (Fer < 15 μg/L)	2.0 (1.0, 2.8)	2.0 (1.0, 3.0)	−0.141	−0.412, 0.130	0.308	331	−0.142	−0.420, 0.136	0.316	225
IDE (sTfR > 8.3 mg/L)	2.0 (1.0, 2.6)	2.0 (1.0, 3.0)	−0.042	−0.300, 0.215	0.747	231	−0.055	−0.327, 0.217	0.692	225
MIS (Fer ≥ 50 μg/L)	2.0 (1.0, 3.0)	2.0 (1.0, 2.0)	0.046	−0.137, 0.229	0.621	231	0.076	−0.115, 0.267	0.435	225

*Hb* haemoglobin, *Fer* serum ferritin, *sTfR* soluble transferrin receptor, *ID* iron deficiency, *IDE* iron deficiency erythropoiesis, *MIS* moderate iron stores, *CI* confidence interval

Analysed with Poisson regression with a log linear link. *P* ≤ 0.05 was considered significant and *P* between 0.05–0.10 to have a tendency toward significance. Model 1 is adjusted for maternal age, parity, HIV, education; total days of morbidity monitored and height; model 2 was adjusted additionally for MUAC, living standards measure (socio economic status), gestational age, and RBC n-3 LCPUFA composition

^a^Values are medians (5^th^, 95^th^) percentiles

Associations of maternal iron status at early pregnancy (< 18 weeks’ gestation) with experiencing respiratory illness during pregnancy assessed with multivariable logistic regression, using all women (n = 225) and women with all visits and $\ge$ 100 days of data (*n* = 155). P between 0.05–0.10 was considered to have a tendency toward significance.

Table 5 shows that anaemic women (Hb < 10.5 g/dL) tended to experience almost double the duration of respiratory illness compared to non, anaemic women with median 15.5 (5.0, 31.0) days compared to median 8.0 (6.0, 12.1) days (β: 0.167, 95% CI: −0.007, 0.342, *p* = 0.060). Respiratory illness episodes in ID women based on ferritin tended to be about double the duration with median 12.0 (7.0, 25.0) days compared to iron sufficient women with median 7.8 (5.3, 13.0) days in the partially adjusted model (β: 0.180, 95% CI: −0.023, 0.384, *p* = 0.082). The ferritin association did not maintain statistical significance in the fully adjusted model. Women with moderate iron stores (ferritin ≥ 50 μg/L) tended to have almost half the duration of respiratory illness compared to women with lower iron stores with median 7.0 (5.0, 10.8) vs 12.0 (6.0, 17.0) days, (β: −0.141, 95% CI: −0.282, 0.000, *p* = 0.051).

**Table 5:** Iron status in association with duration of respiratory or gastric illness during pregnancy in women who experienced these illnesses

			**Respiratory illness**							
	**Duration (days)**		**Model 1**				**Model 2**			
**Exposure**	**Case**	**Control**	**β**	**95% CI**	**P**	**n**	**β**	**95% CI**	**P**	**n**
Anaemia (Hb < 10.5 g/dL)	15.5 (5.0, 31.0)	8.0 (6.0, 12.1)	0.182	−0.012, 0.352	**0.036**	76	0.167	−0.007, 0.342	0.060	75
Anaemia (Hb < 11.0 g/dL)	12.0 (5.0, 20.5)	7.5 (6.0, 12.0)	0.136	−0.026, 0.298	0.098	76	0.125	−0.044, 0.294	0.144	75
ID (Fer < 15 μg/L)	12.0 (7.0, 25.0)	7.8 (5.3, 13.0)	0.180	−0.023, 0.384	0.082	78	0.166	−0.041, 0.373	0.114	76
IDE (sTfR > 8.3 mg/L)	12.0 (7.0, 31.0)	8.0 (6.0, 13.0)	0.196	−0.042, 0.433	0.105	78	0.192	−0.052, 0.435	0.121	76
MIS (Fer ≥ 50 μg/L)	7.0 (5.0, 10.8)	12.0 (6.0, 17.0)	−0.119	−0.254, 0.016	0.084	78	−0.141	−0.282, 0.000	0.051	76
			**Gastric illness**							
	**Duration (days)**		**Model 1**				**Model 2**			
**Exposure**	**Case**	**Control**	**β**	**95% CI**	**P**	**n**	**β**	**95% CI**	**P**	**n**
Anaemia (Hb < 10.5 g/dL)	6.3 (5.0, 19.0)	6.0 (4.0, 9.6)	0.078	−0.117, 0.274	0.422	45	0.067	−0.144, 0.279	0.521	45
Anaemia (Hb < 11.0 g/dL)	7.0 (6.0, 17.5)	5.7 (4.0, 9.3)	0.123	−0.056, 0.303	0.172	45	0.118	−0.077, 0.314	0.226	45
ID (Fer < 15 μg/L)	5.0 (4.0, 19.3)	6.0 (5.0, 10.9)	−0.020	−0.220, 0.181	0.843	47	−0.020	−0.222, 0.182	0.843	46
IDE (sTfR > 8.3 mg/L)	6.0 (5.0, 20.8)	6.0 (4.0, 11.0)	0.114	−0.126, 0.353	0.343	47	0.129	−0.108, 0.367	0.276	46
MIS (Fer ≥ 50 μg/L)	6.3 (5.1, 10.6)	6.0 (4.0, 14.0)	0.008	−0.167, 0.184	0.923	47	0.030	−0.144, 0.204	0.727	46

*Hb* haemoglobin, *Fer* serum ferritin, *sTfR* soluble transferrin receptor, *ID* iron deficiency, *IDE* iron deficiency erythropoiesis, *MIS* moderate iron stores

Analysed with ANCOVA, durations were log transformed before analysis. *P* ≤ 0.05 was considered significant and *P* between 0.05–0.10 to have a tendency toward significance. Model 1 is adjusted for maternal age, parity, HIV, education; total days of morbidity monitored and height; model 2 was adjusted additionally for MUAC, living standards measure (socio economic status), gestational age, and RBC n-3 PUFA composition. Values are medians (25^th^, 75^th^) percentiles

**Fig. 3:**
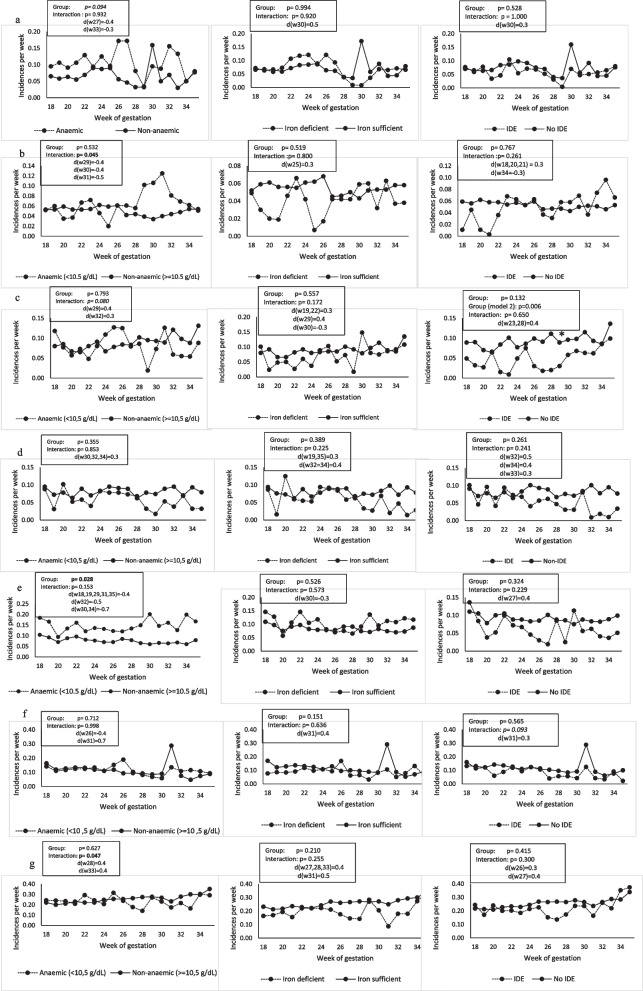
**a**-**g** Longitudinal associations between iron status and morbidity symptoms. Longitudinal association over pregnancy from 18 to 35 weeks’ gestation of anaemia (Hb 8.3mg/L) with a) coughing, b) diarrhoea, c) fever, d) nausea, e) vomiting, f) headache and g) extreme tiredness (n=184). Associations were assessed with linear mixed models adjusted for maternal age, parity, HIV, education; total days of morbidity monitored and height; MUAC, living standards measure (socio, economic status), gestational age and n-3, PUFA status. P ≤ 0.05 was considered significant and P between 0.05 – 0.10 to have a tendency toward significance. IDE, iron deficiency erythropoiesis; fer, ferritin; MUAC, mid upper, arm circumference; PUFA, polyunsaturated fatty acids

Morbidity symptoms over time (18–35 weeks’ gestation) according to iron status are shown in Figs. 3a-g. In Fig. 3a the incidence per week of coughing was borderline higher in the anaemic group compared to the non-anaemic group (*p* = 0.094), being the largest at 27- and 33-weeks’ gestation (d = −0.4 and d = −0.3 respectively), and higher in the non-anaemic group at 30 weeks’ gestation (d = 0.5).

Figure 3b shows that there were more incidences of diarrhoea in the anaemic (Hb < 10.5 g/dL) than non-anaemic groups (interaction *p* = 0.045) at 29-, 30- and 31-weeks’ gestation (d = −0.4, d = −0.4 and d = −0.5, respectively).

In terms of fever (Fig. 3c), there tended to be (interaction *p* = 0.080). More incidences of fever in the anaemic (Hb < 10.5 g/dL) vs non-anaemic group at 25 weeks’ (d = −0.3) and in the non-anaemic vs anaemic group at 29- and 32-weeks’ gestation (d = 0.4 and d = 0.3, respectively). There were more incidences of fever in the non-IDE (iron sufficient erythropoiesis) group at 22, 23-, 26-, 27- and 28-weeks’ gestation (*p* = 0.006, 0.3 < d ≤ 0.4), in the partially adjusted model. The IDE association did not maintain statistical significance in the fully adjusted model (*p* = 0.132).

Anaemic women (Hb < 10.5 g/dL) had more incidences of vomiting throughout pregnancy than non-anaemic women (*p* = 0.028), with a large effect size at 30- and 34-weeks’ gestation (d = 0.7, Fig. 3e). Vomiting decreased over time in IDE and non-IDE women (*p* = 0.016) and tended to decrease in anaemic and non-anaemic (*p* = 0.114) and ID and iron sufficient women (*p* = 0.082).

Headache (Fig. 3f) was borderline higher in the non-IDE group at 31 weeks’ gestation with a small effect size (interaction *p* = 0.093, d = 0.3).

Figure 3g shows more incidences of extreme tiredness in women who were non-anaemic (Hb ≥ 10.5 g/dL) at 28- and 33-weeks’ gestation (interaction *p* = 0.047, both d = 0.4). Extreme tiredness increased over time during pregnancy in anaemic and non-anaemic (*p* = 0.012), ID and iron sufficient (*p* < 0.001) and IDE and non-IDE women (*p* < 0.001).

Longitudinal association over pregnancy from 18 to 35 weeks’ gestation of anaemia (Hb < 10.5 g/dL), iron deficiency (Fer < 15 μg/L) and iron deficiency erythropoiesis (sTfR > 8.3 mg/L) with a) coughing, b) diarrhoea, c) fever, d) nausea, e) vomiting, f) headache and g) extreme tiredness (*n* = 184). Associations were assessed with linear mixed models adjusted for maternal age, parity, HIV, education; total days of morbidity monitored and height; MUAC, living standards measure (socio, economic status), gestational age and n-3, PUFA status. *P* ≤ 0.05 was considered significant and P between 0.05–0.10 to have a tendency toward significance. IDE, iron deficiency erythropoiesis; fer, ferritin; MUAC, mid upper, arm circumference; PUFA, polyunsaturated fatty acids.

## Discussion

Pregnant women who were anaemic and/or ID at early pregnancy in this largely overweight population experienced more and longer durations of infectious morbidity, mostly gastrointestinal, than non-anaemic and iron-sufficient women. Iron is a valuable contributor for normal human immunity and evidence suggests that individuals with ID may be more susceptible to infections than persons with normal iron status [33]. Even so, it should not be disregarded that excess iron can adversely influence immunity. In our population, although with weaker evidence, non-IDE (iron sufficient erythropoiesis) women at early pregnancy showed a tendency to have twice the odds for experiencing any infectious respiratory illness, supported by some evidence for iron sufficient erythropoiesis women to have more fever during pregnancy than IDE women. This finding highlighted a possible detrimental effect of supplementing iron-sufficient women with a relatively high dose of iron.

ID and anaemia are concerns during pregnancy and increase the risk of both foetal and maternal morbidity and mortality [34, 35]. However, supplementation may be ineffective due to poor bioavailability and absorption of oral iron, especially in chronic inflammatory settings with high rates of overweight and obesity, and HIV infection, as in the current study [5, 9]. The newer WHO recommendations for supplementing with a MMS, including vitamin C which could improve absorption by 1.5 to three times, may be a better option for such a population [36, 37].

Our findings agreed that anaemia during pregnancy poses a significant health problem, also with respect to infectious morbidity [35, 38]. Anaemic and/or ID/IDE women in our study tended to have longer durations of respiratory illness and anaemic women experienced more fever and borderline more coughing. Similarly, a study by Smith et al*.* [39] also found that anaemic women had longer hospitalisation durations, as well as increased antenatal admissions. IDA is known to impair immunity by affecting cell, mediated, humoral and nonspecific immunity, as well as the activity of cytokines [40].

Even though women with IDE who contracted respiratory illness were ill for longer, our study found that the odds for women with iron sufficiency (sTfR ≤ 8.3 mg/L) at early pregnancy were about two times higher to experience any respiratory illness compared to women with IDE. This finding provided evidence that iron supplementation in iron sufficient pregnant women may have clinically relevant side effects. Daily supplementation of 60 mg of ferrous iron has been shown to decrease zinc absorption in pregnant women possibly leading to higher susceptibility to infectious morbidity [41, 42] and although zinc status data in pregnant women are unavailable, zinc deficiency are high in South Africa [43, 44]. In rural North West province, South African primary school zinc deficiency prevalence was found to be 75.5% [44]. In addition, iron supplementation may increase the concentrations of free radicals in the intestinal milieu, causing epithelial damage in the intestine [45]. Furthermore, oxidative stress subsequently increases inflammation, which may lead to an immunosuppressed state and increase susceptibility to infections [46].

Our study presented evidence of both iron deficiency and sufficiency to be associated with some morbidities within the context of routine iron supplementation. The complexity of the relationship between iron status, supplementation and infection has been illustrated [33, 47] indicating the need for further evidence to manage iron status optimally.

Strengths of our study are 1) prospective data collection and thus post-exposure outcome (illness) data collection; 2) multiple iron biomarkers adjusted for inflammation and altitude [19]; and 3) several confounders were included which strengthened our analyses. Possible limitations include 1) self-selection bias at recruitment since women were recruited at primary healthcare clinics and then volunteered and agreed to participate at a different setting; 2) loss to follow-up resulted in missing data; and 3) morbidity symptoms were self-reported, and gastric and respiratory illness were scored subsequent to data collection which could have led to misclassification, 4) data on causes of non-infectious gastrointestinal disturbances were not collected, and 5) gastrointestinal side effects from iron supplements increased the risk for misclassification but was mitigated by only scoring an infectious gastrointestinal illness event when symptoms overlapped with at least 3 days of reported fever.

## Conclusion

In conclusion, women with anaemia and/or ID at early pregnancy supplemented daily with iron (~ 55 mg elemental iron) generally experienced more gastric and longer respiratory infectious morbidity than their sufficient counterparts. However, iron sufficient erythropoiesis women at early pregnancy receiving the same daily dose of iron may have twice the risk to experience infectious respiratory illness during pregnancy than women with IDE. Our results highlight the challenge of preventing and managing anaemia and iron deficiency in pregnant woman in South Africa and similar settings. This suggests that it may be prudent to revisit the current strategy of high-dose routine supplementation as recommended by the new WHO guidelines [10].

## Supplementary Information

Supplementary Material 1: Supplementary Table 1. Characteristics of pregnant women at enrolment (< 18 weeks’ gestation) and by morbidity data availability. Supplementary Table 2. Sensitivity analysis using only participants who attended all visits and were monitored ≥100 days: Associations of maternal iron status at early pregnancy with experiencing respiratory or gastric illness at least once during pregnancy (multivariable logistic regression).

## Data Availability

Data will be made available on reasonable request.

## Abbreviations

24-HR: 24-Hour recall
AGP: α1-Acid glycoprotein
BMI: Body mass index
CI: Confidence interval
CRP: C-reactive protein
Fer: Serum ferritin
Hb: Haemoglobin
HIV: Human immunodeficiency virus
ID: Iron deficiency
IDE: Iron deficient erythropoiesis
LSM: Living standards measurements
MIS: Moderate iron stores
MMS: Multiple micronutrient supplement
MUAC: Mid-upper arm circumference
NWU: North-West University
NuPED: Nutrition during Pregnancy and Early Development
n-3 PUFA: Omega-3 polyunsaturated fatty acid
OR: Odds ratio
PUF: Polyunsaturated fatty acid
QFFQ: Quantified food frequency questionnaire
RBC: Red blood cell
SAMRC: South African Medical Research Council
sTfR: Soluble transferrin receptor
WHO: World Health Organisation
