# Supplementary material for: Iron status at early pregnancy is associated with infectious respiratory and gastric illness in women receiving routine iron supplementation: the NuPED prospective cohort
Source: BMC Pregnancy Childbirth. 2025 Jun 4;25:657. doi: 10.1186/s12884-025-07786-8 (PMC12139176; doi:10.1186/s12884-025-07786-8)
Supplement: Supplementary file 1 — Supplementary Material 1: Supplementary Table 1. Characteristics of pregnant women at enrolment (< 18 weeks’ gestation) and by morbidity data availability. Supplementary Table 2. Sensitivity analysis using only participants who attended all visits and were monitored ≥100 days: Associations of maternal iron status at early pregnancy with experiencing respiratory or gastric illness at least once during pregnancy (multivariable logistic regression). [file 12884_2025_7786_MOESM1_ESM.docx]

**Supplementary table 1: Characteristics of pregnant women at enrolment (< 18 weeks’ gestation) and by morbidity data availability**

| **Characteristics** | **Morbidity data**  **>100 days**  **63% (n=157)** | **Morbidity data missing or**  **≤100 days**  **37% (n=93)** | **p** | **Total sample  (n=250)*** |
| --- | --- | --- | --- | --- |
|  | **Median (25^th^, 75^th^)**  **or n (%)** | **Median (25^th^, 75^th^)**  **or n (%)** |  | **Median (25^th^, 75^th^)**  **or n (%)** |
| Age (y) | 27 (24, 32) | 27 (24, 32) | 0.908 | 27 (24, 32) |
| Gestational age (wks) | 14.0 (11.9, 15.9) | 14.7 (12.3, 16.4) | 0.438 | 14 (12, 16) |
| BMI (kg/m^2^) ^#^ | 27.0 (23.1, 30.6) | 25.7 (22.5, 30.7) | 0.541 | 26.3 (23.0, 30.6) |
| Underweight (< 18.5 kg/m^2^) | 6 (3.8) | 2 (2.2) | 0.794 | 8 (3) |
| Normal weight (18.5-24.9 kg/m^2^) | 53 (34) | 36 (39) |  | 89 (36) |
| Overweight (25-29.9 kg/m^2^) | 51 (33) | 30 (32) |  | 81 (33) |
| Obese (≥30 kg/m^2^) | 46 (30) | 25 (27) |  | 71 (28) |
| Ethnicity ^#^ |  |  |  |  |
| Black African | 137 (87) | 82 (89) | 0.459 | 219 (88) |
| Mixed ancestry | 19 (12) | 9 (10) |  | 28 (11) |
| White | 0 (0) | 1 (1) |  | 1 (<1) |
| Indian | 1 (1) | 0 (0) |  | 1 (<1) |
| LSM |  |  |  |  |
| Low (1, 4) | 9 (6) | 8 (9) | 0.098 | 17 (7) |
| Medium (5, 7) | 87 (55) | 61 (66) |  | 148 (59) |
| High (8, 10) | 61 (39) | 24 (26) |  | 85 (34) |
| Highest level of education ^#^ |  |  |  |  |
| Primary school | 7 (5) | 2 (2) | 0.326 | 9 (4) |
| Grade 8 – 10 | 19 (12) | 18 (20) |  | 37 (15) |
| Grade 11 – 12 | 92 (59) | 53 (58) |  | 145 (58) |
| Post-school | 39 (25) | 19 (21) |  | 58 (23) |
| Parity |  |  |  |  |
| Nulliparous | 48 (31) | 26 (28) | 0.536 | 74 (30) |
| Primiparous | 56 (36) | 32 (34) |  | 88 (35) |
| Multiparous | 53 (33) | 35 (38) |  | 88 (35) |
| HIV status |  |  |  |  |
| Positive | 37 (24) | 27 (29) | 0.339 | 64 (26) |
| Negative | 120 (76) | 66 (71) |  | 186 (74) |
| Iron status |  |  |  |  |
| Hb (g/dL)* | 11.8 (10.8, 12.7) | 11.7 (10.6, 12.8) | 0.277 | 11.7 (10.8, 12.7) |
| Fer (μg/L) | 43.2 (21.8, 94.1) | 50.6 (2.8, 100.7) | 0.421 | 47.8 (20.8, 100.8) |
| sTfR (mg/L) | 4.9 (3.7, 6.8) | 4.7 (3.9, 6.1) | 0.771 | 4.8 (3.7, 6.6) |
| Anaemic (Hb <11g/dL)* | 41 (27) | 29 (32) | 0.415 | 70 (29) |
| Anaemia (Hb<10.5g/dL)* | 23 (15) | 20 (22) | 0.178 | 43 (18) |
| ID (Fer <15 ug/L) | 24 (15) | 13 (14) | 0.778 | 37 (15) |
| IDE (sTfR >8.3 mg/L) | 26 (17) | 12 (13) | 0.436 | 38 (15) |
| MIS (Fer ≥50 ug/L) | 70 (45) | 47 (51) | 0.362 |  |
| Inflammatory status |  |  |  |  |
| CRP (mg/L) | 7.0 (3.1, 15.6) | 5.8 (3.1, 12.8) | 0.608 | 6.5 (3.1, 14.1) |
| Elevated CRP (>5 mg/L) | 95 (61) | 54 (58) | 0.703 | 149 (60) |

BMI: body mass index; CRP: C-reactive protein; CI: confidence interval; wks: weeks; LSM: living standards measure; Fer: ferritin; Hb: haemoglobin; ID: iron deficiency; IDE: iron deficiency erythropoiesis; MIS: moderate iron stores; sTfR: soluble transferrin receptor. Differences between groups were analysed with Mann-Whitney, U tests for continuous variables and Chi, square tests for categorical variables. P ≤ 0.05 was considered significant.

# BMI, ethnicity and education missing one data point, *Anaemic (Hb <11g/dL), Anaemia (Hb<10.5g/dL) and Hb (g/dL) missing 7 data points.

**Supplementary table 2: Sensitivity analysis using only participants who attended all visits and were monitored ≥100 days: Associations of maternal iron status at early pregnancy with experiencing respiratory or gastric illness at least once during pregnancy (multivariable logistic regression)**

|  |  |  | **Respiratory illness** | | |  |  | | |  |
| --- | --- | --- | --- | --- | --- | --- | --- | --- | --- | --- |
|  | **Case** | **Control** | **Model 1** | | |  | **Model 2** | | |  |
| **Exposure** | **Fraction (%)** | **fraction (%)** | **OR** | **95% CI** | ***P*** | **n** | **OR** | **95% CI** | ***P*** | **n** |
|  |  |  |  |  |  |  |  |  |  |  |
| Anaemia (Hb<10.5g/dL) | 2/5 (40.0) | 47/130 (36.2) | 1.102 | 0.430, 2.829 | 0.839 | 154 | 1.155 | 0.434, 3.075 | 0.774 | 151 |
| Anaemia (Hb<11.0g/dL) | 2/5 (40.0) | 43/111 (38.7) | 0.648 | 0.294, 1.428 | 0.282 | 154 | 0.624 | 0.269, 1.451 | 0.273 | 151 |
| ID (Fer<15μg/L) | 8/24 (37.0) | 50/135 (37.0) | 1.056 | 0.395, 1.821 | 0.914 | 157 | 0.746 | 0.273, 2.037 | 0.567 | 155 |
| IDE (sTfR>8.3mg/L) | 7/27 (25.9) | 51/132 (38.6) | 0.593 | 0.226, 1.560 | 0.290 | 157 | 0.746 | 0.273, 2.037 | 0.567 | 155 |
| MIS (Fer≥50μg/L) | 29/72 (40.3) | 29/87 (33.3) | 1.262 | 0.633, 2.516 | 0.508 | 157 | 1.256 | 0.609, 2.592 | 0.538 | 155 |
|  |  |  |  |  |  |  |  |  |  |  |
|  |  |  | **Gastric illness** | | |  |  | | |  |
|  |  |  | **Model 1** | | |  | **Model 2** | | |  |
| Anaemia (Hb<10.5g/dL) | 2/5 (40.0) | 28/130 (21.5) | 1.479 | 0.500, 4.376 | 0.480 | 154 | 1.479 | 0.474, 4.608 | 0.500 | 151 |
| Anaemia (Hb<11.0g/dL) | 2/5 (40.0) | 24/111 (21.6) | 1.303 | 0.524, 3.238 | 0.596 | 154 | 1.454 | 0.557, 3.796 | 0.445 | 151 |
| ID (Fer<15μg/L) | 9/24 (37.5) | 27/135 (22.0) | 2.634 | 0.940, 7.380 | 0.066 | 157 | 2.569 | 0.869, 7.596 | 0.088 | 155 |
| IDE (sTfR>8.3mg/L) | 6/27 (22.2) | 30/132 (22.7) | 1.102 | 0.378, 3.218 | 0.859 | 157 | 1.246 | 0.413, 3.760 | 0.679 | 155 |
| MIS (Fer≥50μg/L) | 15/72 (20.8) | 21/87 (24.1) | 0.916 | 0.407, 2.062 | 0.832 | 157 | 0.845 | 0.362, 1.972 | 0.697 | 155 |

Hb: haemoglobin; Fer: serum ferritin; sTfR: soluble transferrin receptor; ID: iron deficiency; IDE: iron deficiency erythropoiesis; MIS: moderate iron stores; OR: odds ratio; CI:

confidence interval.

Associations assessed with multivariable logistic regression, odds ratios and 95% confidence intervals. *P ≤* 0.05 was considered significant and *P* between 0.05 – 0.10 to have a tendency toward significance. Model 1 is adjusted for maternal age, parity, HIV, education; total days of morbidity monitored and height; model 2 adjusted additionally for MUAC, living standards measures (socio, economic status), gestational age, and RBC n-3 LCPUFA composition.
